# Elevated serum autoantibodies against co-inhibitory PD-1 facilitate T cell proliferation and correlate with disease activity in new-onset systemic lupus erythematosus patients

**DOI:** 10.1186/s13075-017-1258-4

**Published:** 2017-03-09

**Authors:** Hui Shi, Junna Ye, Jialin Teng, Yufeng Yin, Qiongyi Hu, Xinyao Wu, Honglei Liu, Xiaobing Cheng, Yutong Su, Mengru Liu, Juanfang Gu, Ting Lu, HaoJie Chen, Hui Zheng, Yue Sun, Chengde Yang

**Affiliations:** Department of Rheumatology and Immunology, Ruijin Hospital, Shanghai Jiao Tong University School of Medicine, No. 197 Ruijin Second Road, Shanghai, 200025 China

**Keywords:** New-onset SLE, PD-1, Autoreactive antibody, T cell activation, Co-inhibitor

## Abstract

**Background:**

Programmed cell death protein 1 (PD-1) plays an important role in immune response regulation as a co-inhibitory signal during T cell activation. However, there is little known about the serum autoantibody profile of PD-1 in systemic lupus erythematosus (SLE), a disease characterized by the breakdown of immune tolerance to self-antigens and an excessive production of autoantibodies. Thus, we aim to investigate the serum levels and function of anti-PD-1 in patients with new-onset SLE.

**Methods:**

Serum levels of anti-PD-1 IgG and IgM isotypes were detected in new-onset SLE patients (*n* = 90), rheumatoid arthritis (*n* = 50), primary Sjogren’s syndrome (*n* = 50), ankylosing spondylitis (*n* = 25), and healthy controls (HC) (*n* = 80) using an enzyme-linked immunosorbent assay (ELISA). The correlation of anti-PD-1 with clinical characteristics and laboratory parameters of patients with new-onset SLE was analyzed. The effects of purified anti-PD-1 IgG from SLE patients on T cell proliferation were measured using flow cytometry.

**Results:**

The data revealed increased levels of anti-PD-1 IgG, but not IgM, especially in new-onset SLE patients, and the positive rate of anti-PD-1 IgG was 30 (33.3%). The level of anti-PD-1 IgG was closely associated with malar rash (OR = 15.773), arthritis (OR = 22.937), serositis (OR = 16.008), hematological (OR = 35.187), renal (OR = 8.306), and neurological involvement (OR = 37.282). Moreover, the serum levels of anti-PD-1 IgG were positively correlated with the SLE disease activity index (SLEDAI) score (r = 0.296, *p* = 0.0046) and the erythrocyte sedimentation rate (ESR) (r = 0.2446, *p* = 0.0201). In vitro examination showed that purified anti-PD-1 IgG obtained from SLE patients enhanced T cell proliferation when co-cultured with dendritic cells (DCs).

**Conclusions:**

The current study indicates, for the first time, that the serum levels of co-inhibitor autoantibodies against PD-1 are elevated in new-onset SLE patients and are associated with disease activity in SLE. Autoantibodies against PD-1, facilitating T cell proliferation, revealed a new insight into the function of negative regulation signals involved in the pathogenesis of SLE.

**Electronic supplementary material:**

The online version of this article (doi:10.1186/s13075-017-1258-4) contains supplementary material, which is available to authorized users.

## Background

Systemic lupus erythematosus (SLE) is a prototypical autoimmune disease that typically manifests with symptoms including rash, arthritis, serositis, nephritis, and neuropsychopathy [1]. It is characterized by a breakdown of immune tolerance to self-antigens, leading to excessive production of autoantibodies [2]. T cells in patients with SLE display altered attributes and are the primary contributors to disease initiation and perpetuation, as they are specialized for activating B cells and dendritic cells as well as secreting cytokines [3]. Although the precise mechanisms that facilitate the initiation of SLE-related pathology remain unclear, there is an indication that, once immune tolerance is compromised, T cells and B cells play important roles in the amplification and perpetuation of the autoimmune and inflammatory responses [4].

Several lines of evidence have demonstrated that insufficient co-inhibition can lead to a loss of self-tolerance and, thus, to autoimmunity, resulting in deleterious inflammation in and destruction of self-tissues mediated by autoreactive T cells and autoantibodies [5]. Our previous study revealed a novel autoantibody against class A scavenger receptors in SLE patients which contributes to the breakdown of immune tolerance by the loss of the ability to clear apoptotic cells [6].

Recently, much attention has been paid to programmed cell death protein 1 (PD-1, CD279), a surface protein which belongs to the immunoglobulin superfamily, and functions as an immunomodulatory molecule. PD-1, binding with its ligand PD-L1, delivers inhibitory signals to negatively regulate the immune response after T cell activation, and thus, maintains the balance of immune tolerance [7]. Upon PD-1 signals, antigen-presenting cells can convert naive CD4+ T cells into Foxp3^+^ induced regulatory T (iTreg) cells [8]. Numerous studies have been reported that PD-1 and PD-L1 play an important role in the pathogenesis of autoimmune diseases in PD-1-deficient murine models, which present lupus-like glomerulonephritis, arthritis, and fatal autoimmune dilated cardiomyopathy [9–11]. In addition, blockade of the PD-1/PD-L pathway could exaggerate or accelerate the development of autoimmune diseases such as diabetes [12] and experimental autoimmune encephalitis [13]. Given the immunomodulatory properties of PD-1 and PD-L, efforts have been made to elucidate the functions of PD-1 and PD-L in SLE patients. Lower PD-1 expression has been found on CD4^+^ T cells in SLE patients and is correlated with PD-1 genotype, suggesting a crucial role of PD-1 in SLE [14, 15]. Moreover, elevated frequency of PD-L1-expressing neutrophils has been reported in patients with SLE, and is correlated with the disease activity and severity of SLE [16]. However, the serum profile of autoantibodies against PD-1 in SLE remains to be determined.

In this study we aimed to investigate the serum levels of autoantibodies against co-inhibitory PD-1 and their impact on T cell proliferation in new-onset SLE patients in vitro.

## Methods

### Patients and healthy controls

The study included 90 consecutive, untreated, new-onset patients with SLE who fulfilled the American College of Rheumatology (ACR) classification criteria for the diagnosis of SLE [17], and 50 patients with primary Sjogren’s syndrome (pSS), 50 with rheumatoid arthritis (RA) and 25 with ankylosing spondylitis (AS) according to the standard diagnostic criteria [18–20]. Finally, samples from 80 sex- and age-matched healthy donors with neither autoimmune nor infectious diseases were collected as healthy controls. Demographic data, clinical characteristics, and laboratory findings of SLE patients were collected. All of the sera samples were stored at -80 °C until use. Disease activity was measured using the SLE disease activity index 2000 (SLEDAI-2 K) [21]. The study was performed in accordance with the Declaration of Helsinki and the principles of Good Clinical Practice. Biological samples were obtained under a protocol approved by the Institutional Research Ethics Committee of Ruijin Hospital (ID: 2016-62), Shanghai, China. All subjects signed written informed consent.

### Production of recombinant human PD-1 extracellular domain

The extracellular domain of the human PD-1 gene (NP_005009.2) Leu24-Gln167 was synthesized by General Biosystems Inc. (Morrisville, NC, USA). The PD-1 extracellular domain gene was fused to the human immunoglobulin (Ig)G1 Fc region at the N-terminus and the 6xHis tag at the C-terminus using the OE-PCR method. The PCR product was digested with BamHI and XhoI, and cloned into the pCDNA3.4-Topo vector (Invitrogen, Grand Island, NY, USA) under the CMV promoter and mouse Ig-kappa signal peptide for high-level secretion. The recombinant PD-1-nFc-His6 was expressed in Expi293 cells by transient transfection using ExpiFectamine reagent (Life Technologies, Carlsbad, CA, USA). After transfection for 3 days, the culture supernatant was collected and the recombinant protein was purified using a protein A column (GE Healthcare, Milwaukee, WI, USA). As a result of glycosylation, the purified PD-1-nFc-His6, as a disulfide-linked homodimer, migrated as an approximately 120 kDa protein in nonreducing SDS-PAGE. The homodimer was incubated with recombinant enterokinase (Novoprotein, Shanghai, China) at 37 °C for 24 h, passed through the protein A column to remove the Fc region, and exchanged to a PBS buffer for storage at 4 °C. The concentration of recombinant PD-1-His6 was determined by bicinchoninic acid (BCA) protein assay (Pierce Biotechnology, Rockford, USA). The recombinant PD-1-His6 consisted of 156 amino acids and, due to glycosylation, migrated as an approximately 35 kDa protein in nonreducing SDS-PAGE.

### Detection of PD-1 autoantibodies

Antibodies against human PD-1 in the sera of SLE patients, pSS patients, RA patients, AS patients and healthy controls were determined by using an enzyme-linked immunosorbent assay (ELISA). Ninety-six-well high-binding plates (Corning, Corning, NY, USA) were coated with recombinant human PD-1 extracellular domain (1 μg per well) overnight at 4 °C in 0.05 mol/L carbonate buffer sodium (PH = 9.6). The antigen-coated wells were washed three times with PBST (PBS plus 0.05% Tween-20) and blocked with PBST containing 5% bovine serum albumin (BSA) for 2 h at 37 °C. The blocking buffer was tapped off and washed as described above before the addition of 100 ul of serum sample (1:100 diluted in 1% BSA). The PD-1 monoclonal antibody (Novoprotein, Summit, NJ, USA) was used as a positive control. Human sera were incubated for 2 h at room temperature followed by incubation with HRP-conjugated goat anti-human IgG and IgM (Abcam, Cambridge, UK) for another 1 h at room temperature. Then, the plates were washed and 100 ul of tetramethylbenzidine (TMB) substrate solution was added. The color development was stopped by the addition of 50 ul of 0.5 M H_2_SO_4_. Absorbance was measured at a wavelength of 450 nm in a microplate reader (Bio-Rad Laboratories, Richmond, CA, USA). All samples and standards were run in duplicates and the readings were performed in duplicates.

### IgG purification

IgG was isolated from the sera of new-onset SLE patients with high levels of anti-PD-1 IgG according to the ELISA assay by protein A agarose (Pierce, Rockford, IL, USA). The isolates were examined for endotoxin contamination using a limulus amoebocyte lysate (LAL) assay (A&C Biological Ltd., Zhanjiang, China). The total protein content was estimated using a BCA assay.

### Purification of specific autoantibodies against PD-1

The purification of specific antibodies was performed according to a previous report [22]. In brief, recombinant human PD-1 was spotted onto a nitrocellulose filter and incubated with the purified IgG from SLE patients. After washing with PBST, the antibodies were eluted with 100 mM of glycine (pH = 2.8). The eluted antibodies were immediately neutralized with 1 M of Tris-HCl (pH = 8.0). Normal human IgG was purchased from Millipore (Darmstadt, Germany). The purified PD-1 antibodies were concentrated with a final concentration of 1 mg/ml. After sterile filtration, the autoantibodies were stored as small aliquot package in -80 °C.

### Preparation of monocyte-derived dendritic cells (DCs)

Peripheral blood monocytes (PBMCs) were isolated from the blood of three healthy volunteers using Ficoll density gradient centrifugation (GE Healthcare). The CD14^+^ PBMCs were isolated by positive selection using CD14 microbeads (Miltenyi Biotec, Auburn, CA, USA) according to the manufacturer’s instructions. The selected cells were cultured in RPMI 1640 containing 10% fetal calf serum (FCS; Gibco, Grand Island, NY, USA), 2 mM L-glutamine, 100 units/ml penicillin, 100 μg/ml streptomycin and supplemented with 20 ng/ml of granulocyte-macrophage colony-stimulating factor (GM-CSF) and 20 ng/ml of interleukin (IL)-4 (R&D Systems, Inc., Minneapolis, MN, USA) in a humidified 5% CO_2_ incubator. On day 7, DCs were stimulated with 50 ng/ml of lipopolysaccharide (LPS) for 24 h. The matured DCs were labeled with anti-human CD80, CD86, and HLA-DR (BD Biosciences Pharmingen, San Diego, CA, USA) and analyzed using a FACS Canto II instrument (BD Biosciences Pharmingen). More than 90% of the cultured cells were triple positive for CD80, CD86, and HLA-DR.

### Preparation of autologous T cells

Autologous T cells were isolated from the PBMC of three healthy volunteers on day 8. The CD3^+^ T cells were isolated by positive selection using CD3 microbeads (Miltenyi Biotec) according to the manufacturer’s instructions. CD3^+^ T cells were cultured in RPMI 1640 containing 10% FCS, 2 mM L-glutamine, 100 units/ml penicillin, 100 μg/ml streptomycin and supplemented with 20 U/ml of IL-2 (R&D Systems, Inc.) and 0.5 ug/ml of mouse anti-human CD3 (BD Biosciences Pharmingen) for 3 d. Activated T cells were stained with PE-labeling anti-human PD-1 antibody (BD Biosciences Pharmingen) and examined using a FACS Canto II instrument.

### T cell proliferation assay

For the proliferation assay, 1 × 10^5^ T cells were labeled with 10 μM of CFSE (Sigma-Aldrich, St. Louis, MO, USA) in 100 μl pre-cold PBS, followed by incubation in 37 °C for 10 min and neutralization with pre-cold fetal bovine serum according to the manufacturer's instructions (Molecular Probes, Leiden, Netherlands). CFSE-labeled T cells were incubated with fresh medium containing purified human PD-1 antibodies from SLE patients or normal human IgG for 1 h at 37 °C. After being co-cultured with 1 × 10^4^ DCs in round-bottom 96-well tissue culture plates (Corning) for 72 h, cells were collected. PerCP Cy5.5-conjugated anti-human CD4 antibody was used for CD4 T cell staining according to the manufacturer’s protocols. Each experiment was preformed, duplicated and analyzed using a FACS Calibur cytometer and analyzed using FlowJo software (Tree Star Inc., Ashland, OR, USA).

### Statistics

For continuous variables, comparisons were carried out using an independent *t* test for two independent samples, a Mann-Whitney *U* test for non-normal data, and a paired *t* test for matched samples. Categorical variables were compared by the chi-square test or Fisher’s exact test. Correlations between groups were evaluated by the Spearman test. Results are expressed as the mean ± standard deviation (SD) of three independent experiments. Multivariate analysis was performed in order to identify the independent factors that are positively associated with the dependent variable. To control for possible confounding parameters, various multivariate logistic regression models were designed. The magnitude of each factor was expressed as an odds ratio (OR). A *p* value less than 0.05 was considered statistically significant. Graphs were drawn using Graphpad Prism (version 6, Graphpad Software, San Diego, CA, USA). Data were analyzed using the IBM SPSS software package for Windows (version 23.0; IBM Corp., Armonk, NY, USA).

## Results

### Characteristics of the study population

A total of 90 patients diagnosed with SLE, 50 patients with pSS (mean age ± SD, 32.39 ± 7.6 years; female/male, 41/9), 50 patients with RA (mean age ± SD, 30.87 ± 8.1 years; female/male, 42/8), 25 patients with AS (mean age ± SD, 30.19 ± 7.1 years; female/male, 20/5) and 80 healthy donors (mean age ± SD, 31.45 ± 8.9 years; female/male, 68/12) were included in this study. There were no significant differences between the groups with regard to age and gender ratio. SLE disease activity was evaluated using the systemic lupus erythematosus disease activity index 2000 (SLEDAI-2 K) [21]. The history, manifestation, and autoantibody profile of SLE was collected for each patient. Demographic and clinical data are summarized in Table 1.

**Table 1:** The clinical characteristics of the new-onset untreated SLE patients (*n* = 90)

Variable	SLE patients
Gender (female/male, n)	76/14
Age (mean ± SD, year)	33.1 ± 9.8
Disease duration (mean ± SD, days)	72.0 ± 53.0
SLEDAI (mean ± SD)	12.9 ± 7.9
Disease activity	
SLEDAI ≤ 8, (n, %)	40, (44.4)
SLEDAI > 8, (n, %)	50, (55.6)
Disease manifestations	
Arthritis (n, %)	41, (45.6)
Rash (n, %)	43, (47.8)
Oral ulcer (n, %)	11, (5.0)
Serositis (n, %)	16, (17.8)
Photosensitivity (n, %)	14, (15.6)
Raynaud’s phenomenon (n, %)	5, (5.6)
Hematological (n, %)	46, (51.1)
Lupus nephritis (proteinuria ≥ 0.5 g/24 h) (n, %)	21, (23.3)
Neuropsychiatric manifestations (n, %)	8, (8.9)
Autoantibody profile	
Anti-dsDNA + (n, %)	75, (83.3)
Anti-Sm + (n, %)	21, (23.3)
Anti-SSA + (n, %)	42, (46.7)
Anti-SSB + (n, %)	13, (14.4)
Anti-U1RNP + (n, %)	29, (32.2)
Anti-Rib-P + (n, %)	15, (16.7)
Anti-nucleosome-A + (n, %)	36, (40.0)

*SLE* systemic lupus erythematosus, *SD* standard deviation, *SLEDAI-2 K* SLE disease activity index 2000

### Increased serum level of co-inhibitory PD-1 autoantibodies in new-onset SLE patients

**Fig. 1:**
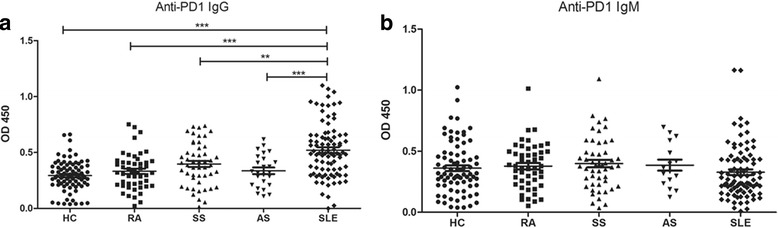
Elevated levels of PD-1 antibodies (IgG isotype) in new-onset SLE patients. The serum levels of PD-1 autoantibodies IgG isotype (**a**) and IgM isotype (**b**) from SLE patients (◆, *n* = 90), pSS patients (▲, *n* = 50), RA patients (■, *n* = 50), AS patients (▼, *n* = 25) and healthy controls (HC, ●, *n* = 80). All standards and samples were run in technical replicates, and readings were performed in duplicates. The optical density (OD) value for each individual is represented as a single point. Values represent the means ± SD. **p < 0.01, ***p < 0.001. *AS* ankylosing spondylitis, *Ig* immunoglobulin, *HC* healthy control, *PD-1* programmed cell death protein, *SS* Sjogren’s syndrome, *RA* rheumatoid arthritis, *SLE* systemic lupus erythematosus 1

For each individual, the optical density (OD) values at 450 nm for anti-PD-1 IgG and IgM were examined using ELISA. As shown in Fig. 1, the OD value of anti-PD-1 IgG in sera from SLE patients was significantly higher than in healthy controls (HC, *p* < 0.001), RA group (*p* < 0.001), pSS group (*p* < 0.01), and AS group (*p* < 0.001) whereas the IgM isotype of PD-1 antibodies showed no significant difference between any two groups. Next, we defined the positive sample as the ratio of the positive and negative optical densities (P/N) greater than 2.1, according to previous work [23]. Among 90 patients with SLE, the positive rate of anti-PD-1 IgG and IgM was 30 (33.3%) and 18 (5%), respectively.

### Association between anti-PD-1 IgG and clinical features of new-onset SLE patients

Table 2 shows the correlation of anti-PD-1 with the clinical parameters of patients with new-onset SLE. The level of anti-PD-1 is closely associated to malar rash (OR = 15.773; 95% CI 3.065–81.186), arthritis (OR = 22.937; 95% CI 4.619–113.9), serositis (OR = 16.008; 95% CI 2.119–120.967), hematological (OR = 35.187; 95% CI 6.459–191.679), renal (OR = 8.306; 95% CI 1.323–52.132), and neurological involvement (OR = 37.282; 95% CI 1.497–928.697). However, no association was observed between anti-PD-1 and the clinical features of discoid rash, oral ulcer, photosensitivity, vasculitis, or Raynaud’s phenomenon (*p* > 0.05). These findings suggest that anti-PD-1 might play a critical role in the occurrence of systematic clinical features.

**Table 2:** Association of anti-PD1 with clinical features of new-onset SLE patients

Clinical manifestations	*p* value	Odds ratio	95% CI
Malar rash	0.001	15.773	3.065–81.186
Discoid rash	0.077	12.688	0.762–211.274
Arthritis	<0.001	22.937	4.619–113.9
Oral ulcer	0.279	3.830	0.336–43.256
Photosensitivity	0.477	4.042	0.086–189.971
Vasculitis	0.216	4.851	0.398–59.109
Raynaud’s phenomenon	0.657	2.266	0.061–84.212
Serositis	0.007	16.008	2.119–120.967
Hematological involvement	<0.001	35.187	6.459–191.679
Renal involvement	0.024	8.306	1.323–52.132
Neurological involvement	0.027	37.282	1.497–928.697

*PD-1* programmed cell death protein 1, *SLE* systemic lupus erythematosus

### Cross-correlation analysis for anti-PD-1 IgG with disease activity

**Fig. 2:**
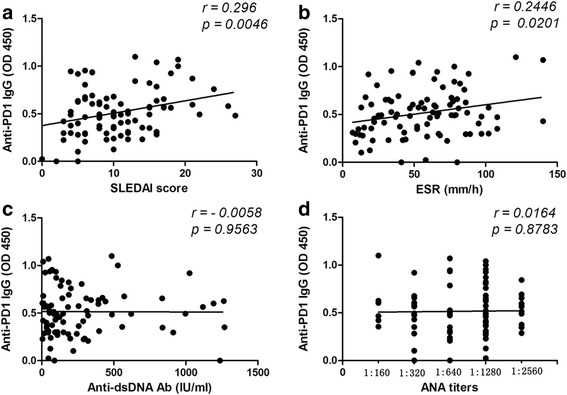
Correlation between anti-PD-1 IgG and SLE disease-related indicators including SLEDAI score (**a**), ESR (**b**), anti-dsDNA (**c**), and ANA (**d**). *ANA* antinuclear antibodies, *ESR* erythrocyte sedimentation rate, *Ig* immunoglobulin, *PD-1* programmed cell death protein 1, *SLEDAI-2 K* SLE disease activity index 2000

**Fig. 3:**
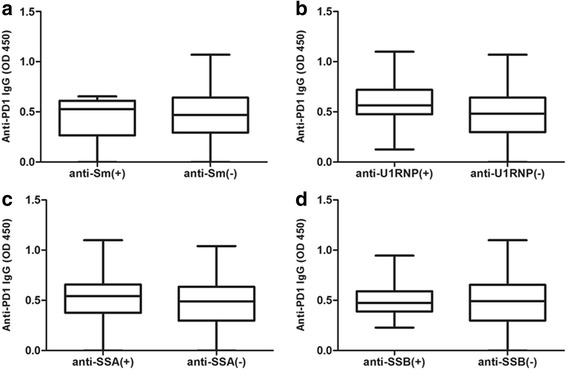
The correlation analysis of serum anti-PD-1 IgG levels between patients with seropositive and seronegative autoantibodies including anti-Sm (**a**), anti-U1RNP (**b**), anti-SSA (**c**), and anti-SSB (**d**). No significant difference was found in the four groups. *Ig* immunoglobulin, *PD-1* programmed cell death protein 1

Next, in order to evaluate the relationship of serum anti-PD-1 levels and disease activity, we first analyzed the correlation between anti-PD-1 IgG, the SLEDAI score, and laboratory parameters including erythrocyte sedimentation rate (ESR), anti-dsDNA, and antinuclear antibodies (ANA). The results show that the serum levels of anti-PD-1 IgG are positively correlated with SLEDAI score (r = 0.296, *p* = 0.0046) and ESR (r = 0.2446, *p* = 0.0201), whereas no statistically significant correlation was observed for anti-PD-1 IgG with anti-dsDNA (r = 0.0398, *p* = 0.9563), ANA (r = 0.0164, *p* = 0.8783) (Fig. 2), as well as C-reactive protein (CRP) and complement C3 and C4 levels (*p* > 0.05, data not shown). Furthermore, we classified SLEDAI scores into four equal groups (group I, SLEDAI = 0–4; group II, SLEDAI = 5–9; group III, SLEDAI = 10–14; group IV, SLEDAI > 14) and found that the serum levels of anti-PD-1 IgG in group I patients were significantly lower than levels in group II (*p* = 0.04) and IV (*p* = 0.001), but not group III (*p* = 0.151) (Additional file 1: Figure S1). Finally, we also analyzed the correlation between anti-PD-1 and other laboratory findings. As shown in Fig. 3, the serum titer of anti-PD-1 IgG is not significantly different between patients with seropositive and seronegative autoantibodies including anti-Sm, anti-U1RNP, anti-SSA, and anti-SSB. These findings indicate that the serum level of autoantibodies against PD-1 might be a potential marker for SLE disease activity.

### Purified autoantibodies against PD-1 from SLE patients promote CD4^+^ T cell proliferation

**Fig. 4:**
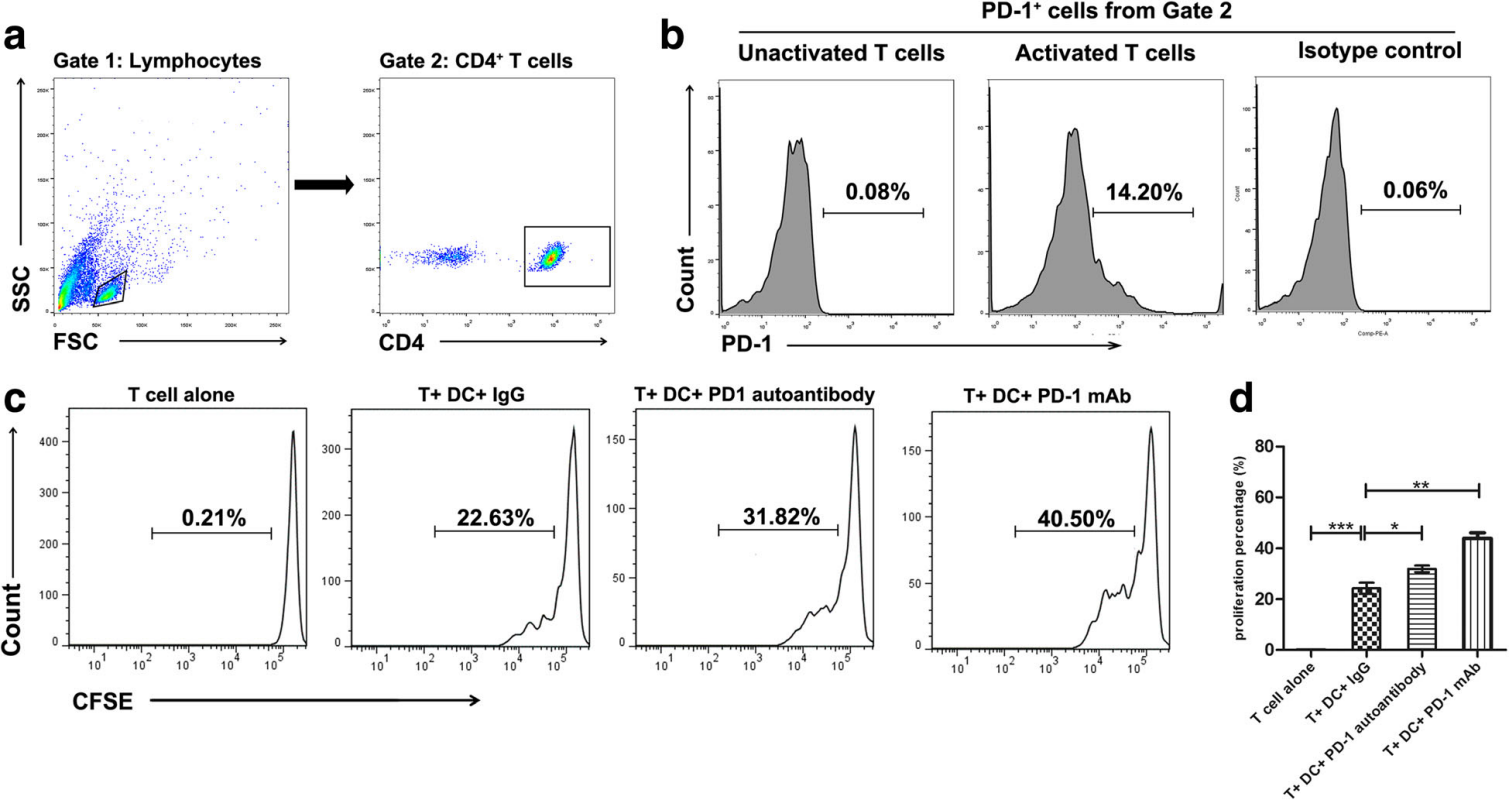
PD-1 autoantibodies increased T cell proliferation. Autologous T cells and DCs were co-cultured for 72 h. **a**, **b** PD-1 expression on T cells activated by anti-CD4 detected by flow cytometry. **c** The division of CFSE-labeled CD4^+^ T cells determined by flow cytometry. The data represent one of three independent experiments. **d** The statistic graph of three experiments. Values represent the means ± SD. *p < 0.05, **p < 0.01, ***p < 0.001. *DCs* dentritic cells, *Ig* immunoglobulin, *mAb* monoclonal antibody, *PD-1* programmed cell death protein 1

Given that co-inhibitor PD-1/PD-L1 is crucial in regulating T cell activation [7], we therefore explored the effect of purified anti-PD-1 from SLE patients on T cell proliferation using a DC and T cell co-culture system. It is well known that PD-1 is expressed on activated T cells. We first examined the PD-1 levels on the surface of CD4^+^ T cells activated by anti-CD3 (0.5 ug/ml) for 3 d. The results showed that PD-1 is expressed after the activation of the T cells, while there was no PD-1 detected on the surface of unstimulated T cells (Fig. 4a, b), which is consistent with previous findings [24].

Next, we purified PD-1 autoantibodies from SLE patients with high levels of anti-PD-1 IgG and carried out a proliferation assay using a T and DC cell co-culture system. The results showed that, compared to the IgG control group, T cell proliferation was enhanced after blocking the PD-1 function with PD-1 autoantibodies from SLE patients and commercial monoclonal PD-1 antibody (Fig. 4c and d). These data indicate that self-reactive autoantibodies against co-inhibitor PD-1 may contribute to the dysregulated T cell hyperproliferation observed during SLE pathogenesis.

## Discussion

Recent studies have demonstrated that immune checkpoints, such as the PD-1 pathway, are vital regulatory pathways for maintaining the homeostasis and tolerance of the immune system [5, 25]. Selective blockade of immune checkpoints reinforce antitumor immunity, in turn resulting in autoimmune diseases such as SLE and rheumatoid arthritis [25, 26]. PD-1, a member of the B7 receptor family, has been reported to be expressed mainly on activated T cells, B cells, and monocytes [24, 27, 28]. After binding with PD-L1, PD-1 delivers a negative signal which inhibits immune responses via downregulating T cell activation, and, thus, affecting peripheral T cell tolerance.

PD-1 is the most studied co-inhibitory receptor in SLE. The polymorphism of PD-1 has been established to be associated with susceptibility to SLE [29–31]. In particular, T cell proliferation was increased in homozygous PD1.3 (A/A) SLE patients, while it was significantly reduced in heterozygous and wild-type (G/G) patients and healthy controls [15]. Previous studies revealed that a lack of PD-1 or PD-1 blockade by anti-PD-1 commercial antibodies result in an exacerbation of T cell-mediated immunopathology in various disease models, including SLE. Furthermore, PD-1 knockout mice developed lupus-like autoimmune symptoms spontaneously and had significantly increased levels of immunoglobulin [9]. There was an interesting finding that T cell proliferation could be increased by adding sera from active SLE patients with unknown mechanism [15]. Our results reveal, for the first time, anti-PD-1 antibody in sera from SLE patients promoting the T cell proliferation, indicating a pathogenic role for anti-PD-1 antibodies in SLE.

Recently, the anti-PD-1 antibody has been suggested as a new serological marker for type 1 autoimmune hepatitis [32]. However, there is still little known about the expression level and function of self-reactive autoantibodies against PD-1 in SLE patients. In the present study, autoantibodies against PD-1 IgG and IgM were found in SLE patients (33.3% and 5%, respectively) specifically. In addition, our data also indicated that serum levels of anti-PD-1 IgG are positively correlated with specific clinical symptoms and disorders including malar rash, arthritis, and serositis, as well as multiple organ involvement including hematologic disorders, renal diseases, and neurological dysfunctions (as shown in Table 2). Our results also revealed that the level of anti-PD-1 was closely associated with the disease activity indicators of SLE patients including the SLEDAI score and ESR. Furthermore, when we stratified patients according to their SLEDAI score, we found that the levels of anti-PD-1 IgG in new-onset SLE patients with SLEDAI scores of 0–4 were significantly lower compared with patients with SLEDAI scores of 5–9 and >14. These results suggest that serum levels of anti-PD-1 IgG might be a helpful indicator to evaluate the disease activity of SLE patients.

The role of the PD-1 pathway in maintaining immune tolerance at the level of DC-T cell interactions has been well established [33], and persistent activation of self-reactive CD4^+^ T cells is a pathogenic hallmark of autoimmune diseases such as SLE [3]. Considering the elevated levels of PD-1 autoantibodies in SLE patients, we further examined the function of these self-reactive antibodies extracted from the sera of SLE patients in promoting CD4^+^ T cell proliferation. Our results showed that the proliferation of DC-activated CD4^+^ T cells was enhanced after incubation with PD-1 autoantibodies, indicating that the co-inhibitory PD-1 pathway is blocked by the autoantibody against PD-1 pathologically in SLE patients. Our data demonstrate that the dysfunction of PD-1, beyond the polymorphism, owing to the self-reactive autoantibodies.

It has been demonstrated that PD-1 knockout in C57 BL/6 mice resulted in the development of lupus arthritis and glomerulonephritis, which indicated the important role of PD-1 in maintaining the homeostasis of autoimmunity [12]. Moreover, lower expression of PD-1 on PBMCs and CD4 + CD25+ T cells have been found in SLE patients [14, 15]. Combined with our current results, antibodies against PD-1 might accelerate the severity of lupus in vivo by blocking the biological function of the PD-1 signaling pathway. Controversially, in vivo blockade of PD-1 receptor in a lupus murine model (NZB/WF1) has been shown to delay the disease progression [34–36], and might, owing to the high affinity and doses of commercial PD-1 antibodies administered to mice, result in the dissolution of T cells instead of the inhibition of T cells function.

Despite PD-1 acting as a negative signal for T cell activation and proliferation, it has been shown that PD-1 signaling is also required for the continued maintenance of functional Tregs that may control autoimmunity in lupus-prone mice models [35]. Besides, the expression level of PD-1 on Tregs was decreased remarkably in SLE patients compared with healthy donors [14]. The PD-1 pathway and Tregs are both crucial for attenuating immune responses by regulating T cell activation. Confusingly, proportions of Tregs in SLE patients were not fully confirmed. Numerous studies have found decreased frequency and defective regulatory function of CD4^+^ CD25^+^ Tregs in SLE patients, which is inversely correlated with disease activity (SLEDAI scores) and serum levels of autoantibody [37–40]. However, unaltered or increased frequency of Tregs, positively correlated with the disease activity in SLE patients, has been demonstrated in some other studies [41, 42]. Thus, more research needs to be done to elucidate the role of PD-1 and Tregs in SLE.

In general, the function of PD-1 autoantibodies in vivo remains complicated and additional animal studies are required to elucidate the contribution of PD-1 autoantibodies on immunoregulation function including Tregs in SLE.

## Conclusions

Autoantibodies against PD-1 were significantly high in the sera of new-onset SLE patients and exacerbated T cell proliferation via blocking the function of immune checkpoint PD-1, suggesting that PD-1 autoreactivity might be a novel pathogenesis contributing to the development of SLE.

## Additional file

Additional file 1: Figure S1. Serum levels of anti-PD-1 IgG in new-onset SLE patients classified into four equal groups according to the SLEDAI score. Values represent the means ± SD. ****p* < 0.001. ***p* < 0.01. **p* < 0.05. *ns* no significance. (TIF 402 kb)

## Abbreviations

ANA: Antinuclear antibodies
AS: Ankylosing spondylitis
DCs: Dentritic cells
ELISA: Enzyme-linked immunosorbent assay
ESR: Erythrocyte sedimentation rate
Ig: Immunoglobulin
IL: Interleukin
PBMCs: Peripheral blood monocytes
PD-1: Programmed cell death protein 1
PD-L: PD-1 ligands
pSS: Primary Sjogren’s syndrome
RA: Rheumatoid arthritis
SLE: Systemic lupus erythematosus
SLEDAI-2 K: SLE disease activity index 2000
Tregs: Regulatory T cells
