# Supplementary figures and images for: Elevated serum autoantibodies against co-inhibitory PD-1 facilitate T cell proliferation and correlate with disease activity in new-onset systemic lupus erythematosus patients
Source: Arthritis Res Ther. 2017 Mar 9;19:52. doi: 10.1186/s13075-017-1258-4 (PMC5343377; doi:10.1186/s13075-017-1258-4)

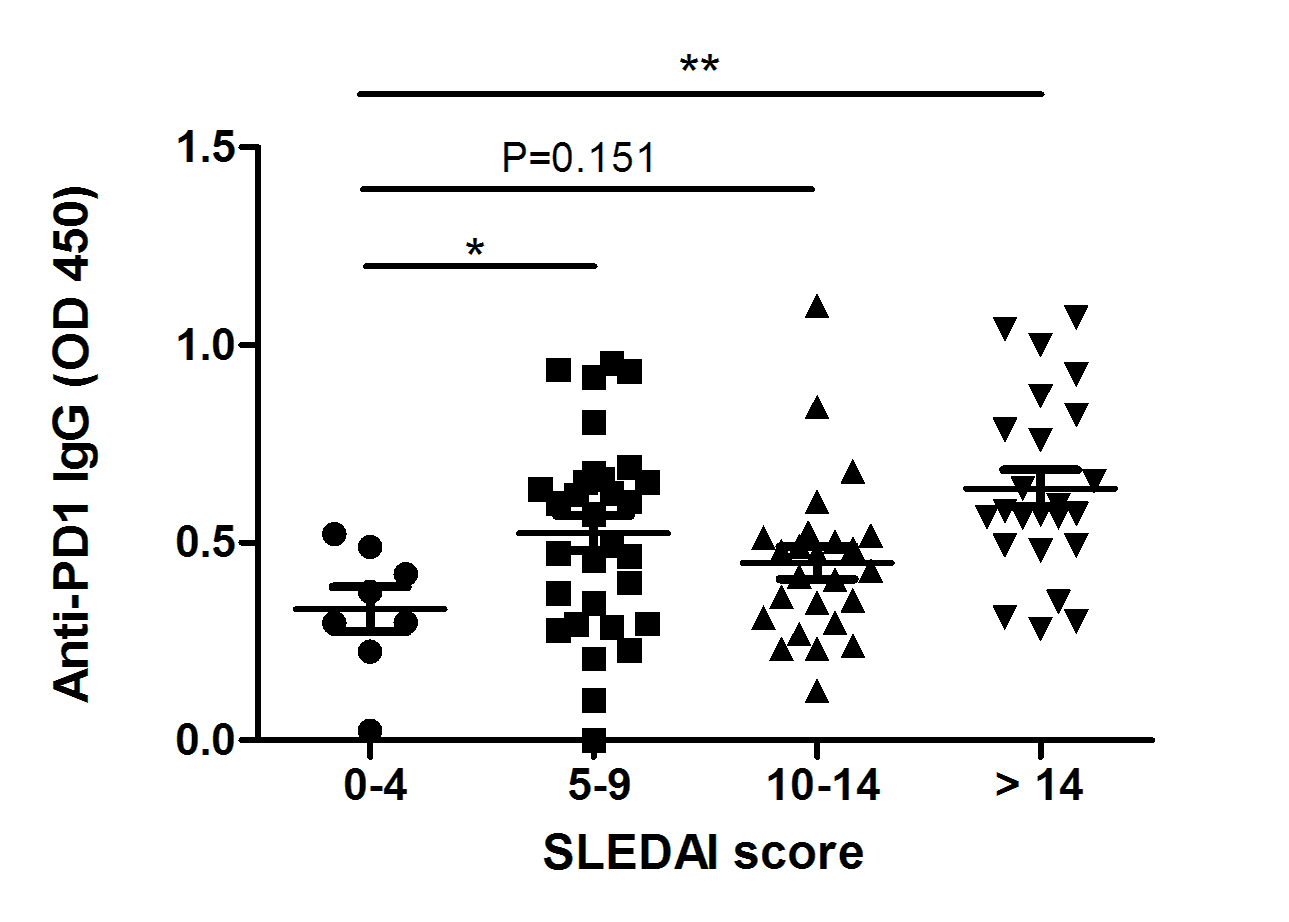

Supplement: Additional file 1: Figure S1. — Serum levels of anti-PD-1 IgG in new-onset SLE patients classified into four equal groups according to the SLEDAI score. Values represent the means ± SD. ***p < 0.001. **p < 0.01. *p < 0.05. ns no significance. (TIF 402 kb) [file 13075_2017_1258_MOESM1_ESM.tif]
